# Recent Advances in Cognitive-Behavioural Therapy for Eating Disorders (CBT-ED)

**DOI:** 10.1007/s11920-024-01509-0

**Published:** 2024-05-08

**Authors:** Glenn Waller, Jessica Beard

**Affiliations:** Clinical and Applied Psychology Unit, Department of Psychology, University of Sheffield, Cathedral Court, 1 Vicar Lane, Sheffield, S1 2LT UK

**Keywords:** Eating disorders, Cognitive-behaviour therapy, Effectiveness, Accessibility, Duration, Review

## Abstract

**Purpose of Review:**

Eating disorders require more effective therapies than are currently available. While cognitive behavioural therapy for eating disorders (CBT-ED) has the most evidence to support its effectiveness, it requires substantial improvement in order to enhance its reach and outcomes, and to reduce relapse rates. Recent years have seen a number of noteworthy developments in CBT-ED, which are summarised in this paper.

**Recent Findings:**

The key advances identified here include: improvements in the efficiency and availability of CBT-ED; expansion of applicability to younger cases across durations of eating disorder; and new methodologies.

**Summary:**

There have been important recent advances in the field of CBT-ED. However, it is important to stress that there remain gaps in our evidence base and clinical skills, and suggestions are made for future research and clinical directions.

## Introduction

Eating disorders come in a number of forms, characterised by psychologically-driven unhealthy patterns of eating (e.g., severe restriction; emotionally-driven eating or avoidance of food; overeating and binge-eating; purging behaviours). Diagnoses include anorexia nervosa, bulimia nervosa, binge-eating disorder, other specified feeding and eating disorder (OSFED; including atypical cases), and avoidant/restrictive food intake disorder (ARFID) [1]. Eating disorders have a relatively high mortality rate among psychiatric disorders, due to physical sequelae and mental health issues [2, 3].

Cognitive-behavior therapy for eating disorders (CBT-ED) is one of the most strongly evidence-based therapies for this group of problems [4, 5]. CBT-ED encompasses a range of protocols that have demonstrated effectiveness with eating disorders [6–9]. It is a widely used approach to non-underweight eating disorders among adults (e.g., bulimia nervosa, binge-eating disorder; OSFED), and its limited outcomes have been shown to be no different from other evidence-based therapies for anorexia nervosa in adults [10, 11]. It has had a more limited evidence base among children and young people, so has not been the first choice in working with that age group [4, 5]. There is evidence for the CBT-ED approach in face-to-face work, group, and guided self-help formats. Central elements involve teaching the patient that their emotional and cognitive reactions to food and eating are inaccurate, and addressing issues of body image. Recent reviews and meta-analyses [12, 13] have confirmed the effectiveness of CBT-ED in a range of settings, including in everyday clinical practice.

However, the relative success of CBT-ED should not blind us to its limitations and the need for further research and development in the field. Recovery rates are far from perfect, particularly for anorexia nervosa, and relapse rates are substantial enough to merit concern [13]. It is important to consider recent developments in the field of CBT-ED so that we can determine where we have improved and where there is more to do. This review will therefore focus largely on developments over the past three years, to update on a similar review [14]. It will consider:

- efforts to improve efficiency and availability of CBT-ED;
- applicability to younger cases and those with different durations of eating disorder;
- new methodologies; and
- remaining gaps in our evidence base and clinical skills.

## Improvements in Efficiency and Availability of Therapies

Waiting lists for treatment of eating disorders can be (and often are) distressingly long, and they lengthened during and after the COVID-19 pandemic. Longer waiting times carry their own distress for the patient, enhancing their sense of helplessness, impacting on medical and psychological risks, and further worsening quality of life. Enhancing accessibility of therapies to patients and the efficiency of those therapies each contribute in an inter-linked way to addressing long waiting times effectively.

### More Efficient Therapies

The motivation to improve efficiency of therapies can be seen as being that we achieve better patient turnover – faster, effective treatments enable clinicians to see more patients without sacrificing the benefits of CBT-ED. In recent years, there have been a number of such developments. Ten session CBT-T for non-underweight patients [15] has demonstrated significant reductions in global eating disorder psychopathology with large effect sizes. Furthermore, CBT-T has resulted in large positive effects on bingeing, purging, depression and quality of life, and a moderate effect on anxiety. These outcomes are all similar to those of 20-session CBT for non-underweight patients [16]. Such briefer individual and group therapies [17•] can enhance patient access to effective treatment. Similarly, group Dialectical Behaviour Therapy (with its focus on behavioural, cognitive, and emotional change) can be as effective in half the time [18, 19]. Guided self-help has also demonstrated important steps towards greater efficiency over a limited therapy period [20], remaining more effective than pure self-help [21, 22], and performing better than other internet-based approaches [23]. There are also early signs that including CBT-ED elements in apps and in single-session interventions can engage patients better and encourage greater change during subsequent therapy [24, 25•, 26]. However, other efforts to enhance efficiency have not always proven effective. For example, researchers have tested the potential to add ‘just in time adaptive interventions’ to existing therapies, but that has not yet proven effective [27].

### More Accessible Therapies

The accessibility of therapy has also been addressed in recent research, showing that people can avail themselves of CBT-ED in a more timely fashion where services are configured to ensure that early identification and entry to treatment are priorities. This became an important issue during the COVID-19 pandemic, where accessibility was enhanced via online access to CBT-ED [28, 29]. Indeed, our learning about the viability of provision of online CBT-ED treatment is one of the unexpected outcomes of the COVID-19 pandemic.

More planful initiatives in accessibility are worthy of particular consideration. In the UK, for example, the First Episode Rapid Early Intervention for Eating Disorders (FREED) programme has been devised to ensure rapid access to treatment for younger adult patients and those with a relatively recent onset [30]. Duration of an untreated eating disorder and waiting times were substantially shorter for FREED patients than for ‘treatment as usual’ patients [31], and the programme has yielded positive outcomes when using the briefer, efficient forms of CBT-ED therapies outlined above [32]. However, it should be stressed that low completion rates [33] mean that these FREED results should only be treated as indicative at present, especially as implementation of the scheme has its challenges [34].

Given the economic costs of eating disorders, including work-related losses, a further promising line of enquiry is whether CBT-ED can be delivered effectively via workplaces. This approach has been shown to be feasible and to have medium to large positive effect sizes on measures of pathology (i.e., eating pathology, depression, anxiety, binge-eating) and on work-related impairments [35].

## Temporal Factors: Age of Patients and Duration of Eating Disorder

Guidelines [4, 5] usually default to family based treatment (FBT) as the first option when working with children and adolescents (apart from those with ARFID), given the substantial evidence base for FBT. However, there are circumstances in which CBT-ED should be considered as an alternative (e.g., where FBT has not proven acceptable to the young person or their families; where FBT has not been fully effective), as it has been demonstrated to improve eating attitudes, clinical impairment, and weight gain in such cases [36]. CBT-ED has a role in working with younger people [37], as shown by more recent evidence that young people with eating disorders show substantial weight gain and reduced scores on eating disorder and general psychopathology with CBT-ED, including longer (20–40 session) and briefer (10 session) versions [37–40].

Duration of the individual’s eating disorder has been an issue addressed in the eligibility criteria for the FREED programme. However, there is less clarity in efforts over the past decade to define ‘severe and enduring eating disorder’ (SEED) or ‘severe and enduring anorexia nervosa’ (SEAN) in a meaningful and useful way. For example, SEAN has been labelled as being of at least three years duration [41] and at least seven years duration [40], even within the same team of clinical researchers. Nor is there consistency on the ‘severity’ criterion. Obviously, without any consistent criteria for such a label, the utility of labels such as SEED and SEAN remains limited. However, there is also evidence that the duration of the disorder is less relevant clinically. It has previously been shown that duration of anorexia nervosa is not related to outcomes when using CBT-ED oriented to full recovery [42, 43]. Recent evidence supports this conclusion for CBT-ED for young people [44••]. Overall, the evidence from systematic reviews and meta-analyses does not support any such role for severity or duration in determining treatment-seeking. Nor does it limit the outcome of therapies broadly [45, 46], or in the domain of CBT-ED more specifically [47].

The final temporal factor to reiterate is the most well-established predictor of outcomes in therapy for eating disorders – the level of change achieved over the first few sessions of therapy. This temporal predictor has been well-established as relevant to outcomes [48, 49]. It has been supported by more recent updates to this meta-analysis [50], where the majority of interventions were CBT-ED. This ‘early change predicts therapeutic outcome’ heuristic extends to guided self-help forms of CBT-ED [51].

## New Methodologies in CBT-ED

There have been several developments in recent years in the technology of CBT-ED, meriting future implementation. These include developments in the exposure therapy element, CBT for ARFID, potential treatment-matching for trauma in eating disorders, and measurement of clinician competence.

### Inhibitory Learning in Exposure Therapy

Inhibitory learning has been suggested as an approach to exposure therapy [52–54], and has been incorporated into brief, effective CBT-ED [9, 15, 55]. The inhibitory learning model states that in addition to repeated exposure to the feared stimulus, it is also important for the individual to learn more generalised safety – disconfirming expectations that the feared stimulus is threatening [56]. Recent research in anorexia nervosa has shown that this element of CBT-ED is associated with rapid learning of safety relating to food, particularly considering between-session exposure [57] and when using virtual food exposure [58]. A focus on inhibitory learning is well-justified in future research, and the guide provided by Melles and colleagues [56] for applying inhibitory learning to anorexia nervosa is recommended for those who are new to this way of implementing CBT-ED.

### CBT-ED for ARFID

Building on work from before DSM-5 brought in the wider diagnosis of avoidant/restrictive food intake disorder (ARFID) [59], early developments in CBT-ED for ARFID showed that exposure-based approaches are effective in addressing different presentations of this disorder [8, 60•]. There has been some advance in this field, with early evidence that this approach can be effective among those with gastroenterology issues alongside ARFID [61] and those with Rumination Syndrome [61]. There are also suggestions of approach-specific outcomes when using CBT-ED with different ARFID presentations [62], but we are still awaiting the outcome of more definitive studies in the field of CBT-ED for ARFID. However, there is now a set of validated measures to support the evaluation of ARFID and the use of CBT-ED in such cases, rather than relying on other, less relevant eating disorder measures [63, 64].

### Potential Treatment Matching in Cases Involving Trauma

Matching treatments to individual characteristics is a very undeveloped area in the field of eating disorders, meaning that the most effective approach for most people is to use the overall most effective treatment – often CBT-ED in adults. One area where recent developments offer a potential evidence-based approach to treatment matching is where the eating disorder presentation is associated with a trauma history. For example, there can be a long-term benefit (but not an immediate one) of adding eye movement desensitization and reprocessing (EMDR) to CBT-ED among patients with anorexia nervosa and a history of childhood maltreatment [65••]. On average, those treated with CBT-E plus EMDR reported a greater reduction in eating disorder specific psychopathology and greater improvements in terms of weight recovery, compared to those treated with CBT-E alone. Similarly, individuals with an eating disorder and a childhood trauma history respond better to compassion-focused therapy than to routine CBT-ED, but in the longer term rather than immediately (and not showing an impact on exercise levels) [66, 67]. In contrast, when considering post traumatic stress disorder (PTSD), routine CBT-ED versus an integrated CBT for eating disorders and PTSD did not differ in their outcomes, either immediately or in the longer term [68]. So, the recent evidence supports modifying approaches for eating disorders where there is a history of trauma, but not when there is comorbid PTSD.

### Measurement of Clinician Competence in Delivering CBT-ED

The Cognitive Therapy Scale-Revised [69] is commonly used to determine clinician competence in delivering CBT for anxiety and depression, but is less reliable and valid when working with other disorders. Earlier efforts to deliver measures of such competence [70, 71] have been limited to CBT-E [72] and have been limited methodologically. This has led to the development of the Cognitive Behaviour Therapy Scale for Eating Disorders (CBTS-ED), based more specifically on observation of clinicians working with patients with eating disorders [73], though further validation work is needed to support its use.

## Future Directions in CBT-ED Research: What Are the Gaps that We Still Need to Fill?

Although we have pointed to a number of positive developments in CBT-ED over recent years, we cannot be complacent about the current position. Clearly, given its outcomes and relapse levels, CBT-ED is far from perfect. Therefore, we need to improve the core protocols for eating disorders – particularly for underweight patients. However, there are more specific areas where clinical research is needed to enhance specific elements of CBT-ED or to support their use at all.

### Digital Interventions

While digital interventions are less expensive than clinician-delivered approaches, they lack strong evidence of effectiveness that would support their widespread use where there are better-evidenced approaches [74]. There is further research planned to test the potential use of such interventions [75], but we await convincing evidence of the outcomes.

### Weight Management

Outcomes with weight loss remain limited and relatively short term [76, 77]. While several such trials are under way [78], it is not yet clear that we will see the hoped-for advances in the field of CBT for weight management.

### Treatment-Matching Heuristics

We have seen some early suggestions (above) that CBT-ED approaches might be matched to trauma histories. However, there are many other factors that might be used in treatment matching. In particular, strategic clinical responses to the patient’s clinical progress remain a good option [36]. For example, Grilo and colleagues [79] have supported the use of CBT-ED where other approaches have not been effective.

As noted above, there is limited evidence that pre-treatment characteristics predict treatment outcome. However, for CBT-ED, there are some recent suggestions that higher levels of early dietary restraint and overvaluation of weight and shape predict reduction in eating pathology (though not abstinence from binge eating) [80]. Similarly, fast or slow early change in CBT-ED for binge-eating can be linked to greater levels of symptom return than moderate levels of early change [81], with some role for mood [82]. These two sets of findings support the conclusion that a combination of baseline scores and early trajectory of change might combine to predict outcomes [83], and this potential heuristic certainly merits further clinical research.

### Adaptations of Therapy for Specific Groups

One area where there is promising evidence is in the treatment of eating disorders in those who are **gender diverse**. Studies of CBT-ED outcomes among such individuals have been relatively sparse to date [84], but recent work has supported its acceptability and preliminary effectiveness with individuals who self-identify as being from gender minorities [85•]. Therefore, this is a promising area for further expanding the reach of CBT-ED to support a group who have been under-served in the treatment literature, despite relatively well-identified levels of eating and body concerns [86, 87].

**Neurodiversity** is the other current area where recent clinical awareness of the problems involved are not well matched with treatment evidence. Of course, the diagnosis of problems such as autistic spectrum disorders and attention deficit hyperactivity disorder needs to be very cautiously undertaken in the context of eating disorder phenomena (starvation, dissociation) and comorbidity (e.g., obsessive compulsive disorder; substance misuse). However, in cases where there is clear neurodiversity, adaptations should be considered that help patients to engage in and benefit from treatment. Methods that might help in such circumstances (e.g., the PEACE pathway) have been suggested [88], though the benefit of these methods has yet to be established within CBT-ED approaches.

## Conclusions

There have been substantial developments in CBT-ED in the past three years, building on existing evidence of effectiveness and reach. However, as stated above, we are a long way from perfection, so we should be thinking of broader enhancement of CBT-ED, rather than simply making relatively small changes. That might mean studies that involve active elements of different forms of CBT-ED, enhancing the use of other techniques that are more commonly associated with third wave therapies [89], learning from other therapies, and considering service user perspectives on what is effective at different stages in the therapy.

Finally, from the recent literature, it is clear that we need more concrete evidence of effectiveness in ARFID, and better overall outcomes in anorexia nervosa. It will be important to determine the effectiveness of CBT-ED in conjunction or in contrast with FBT and other approaches for children and young people. We also need to ensure that CBT-ED is delivered competently, given existing evidence of clinicians reporting weak adherence to any protocol when working with eating disorders [90].

## Data Availability

No datasets were generated or analysed during the current study.
